# Endoscopic ultrasonography-guided single balloon-occluded gastrojejunostomy bypass: A case report

**DOI:** 10.1055/a-2414-7602

**Published:** 2024-10-14

**Authors:** Kai-Xuan Wang, Ping-ping Zhang, Ting Yang, Yu Zhang

**Affiliations:** 1 12520 Department of Gastroenterology, Changhai Hospital, Shanghai, China

**Fig. 1:**
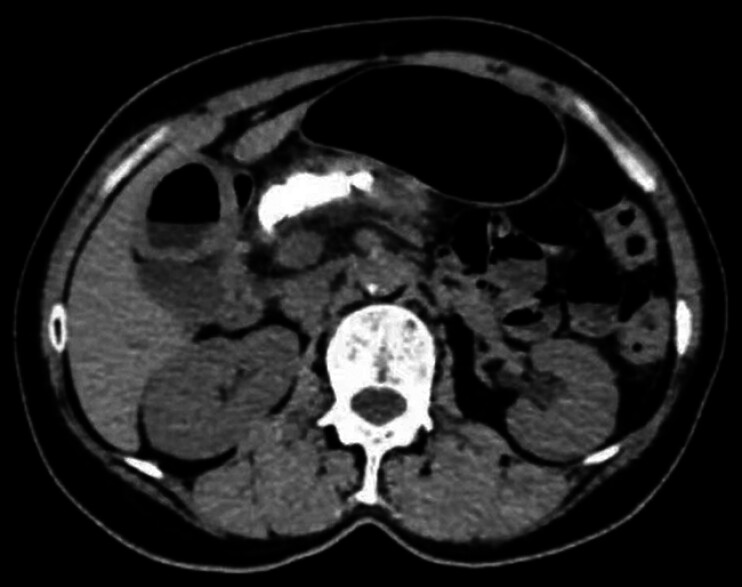
Computed tomography (CT) image showed pancreatic cancer, stenosis of the common bile duct and pancreatic duct, with the stenosis impinging on the horizontal part of the duodenum, and obstruction of the superior mesenteric artery and vein.

**Fig. 2:**
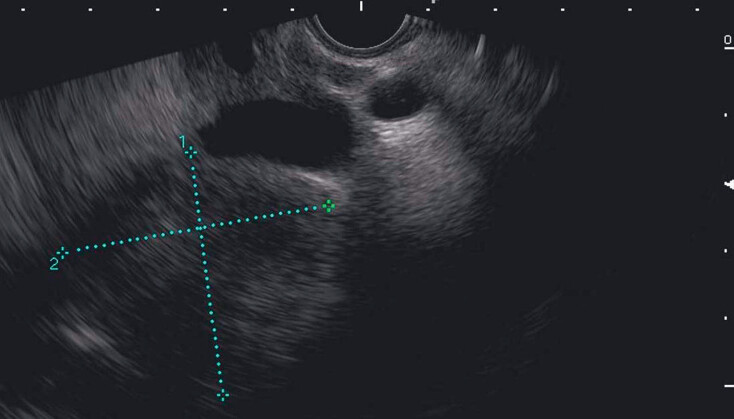
Endoscopic ultrasound findings were consistent with those of CT.

**Fig. 3:**
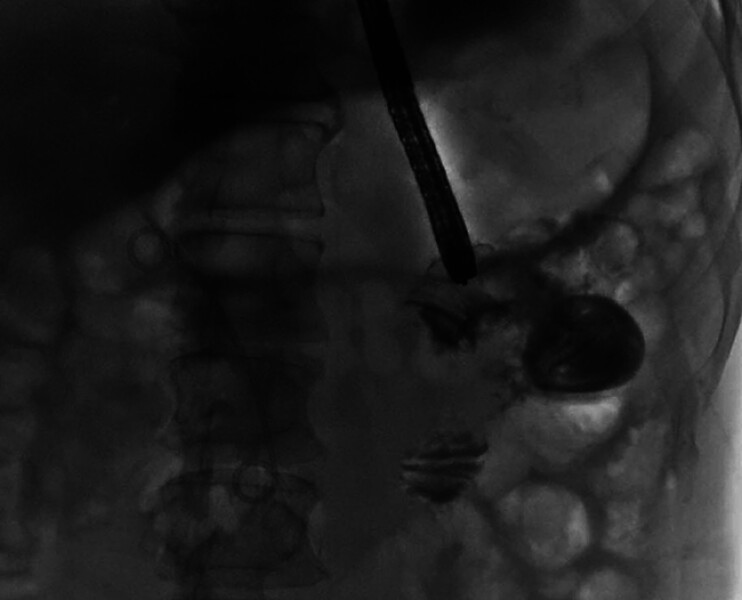
Gastro-enterography image showed narrowing of the duodenum.

A 67-year-old man presented at our institution with yellow staining of his skin and sclera for more than 1 month. Laboratory examination showed high concentrations of total bilirubin and carbohydrate antigen 19–9. Contrast-enhanced computed tomography (CT) of the abdomen showed pancreatic cancer with common bile duct and pancreatic duct stenosis. The horizontal part of the duodenum was invaded by the stenosis, obstructing the superior mesenteric artery and vein ( Fig. 1 ). The findings of endoscopic ultrasound (EUS) were consistent with those of CT ( Fig. 2 ), and gastro-enterography showed a narrowing of a 2.8-cm length of the duodenum ( Fig. 3 ).

**Fig. 4:**
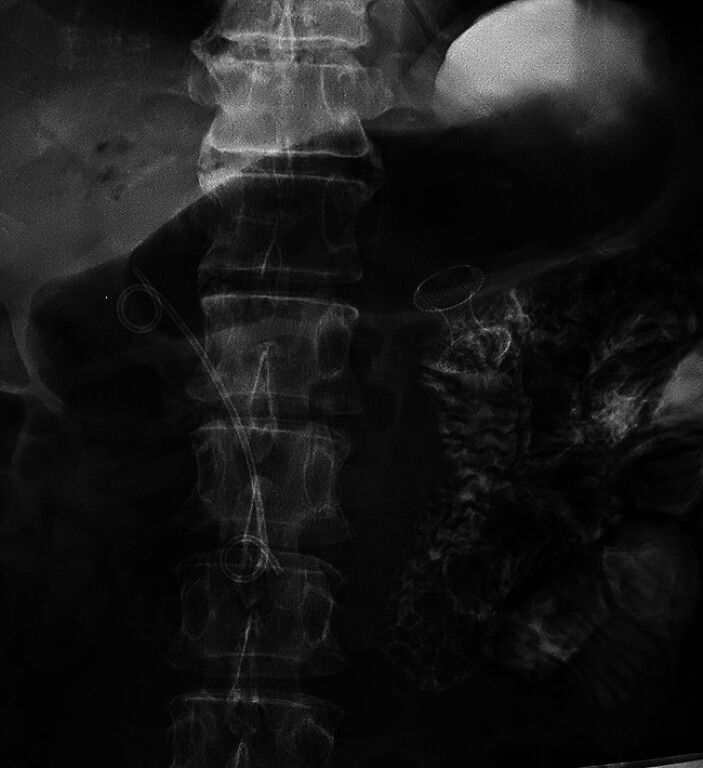
Lumen-apposing metal stents were successfully deployed through the gastric wall into the jejunal wall.

Implementing a single balloon-assisted technique and under general anesthesia, a gastroenterostomy bypass was created under EUS guidance. A 0.025-inch guidewire (Olympus Medical Systems, Tokyo, Japan) was inserted through the accessory biopsy channel of an Olympus GIF-H 260 across the obstruction to the jejunum, after which the endoscope was removed, leaving the guidewire in place. A balloon (Micro-Tech, Nanjing, China) filled with 40 mL of air was inserted over the guidewire into the distal small bowel under X-ray fluoroscopy assistance. Saline and contrast with methylene blue were then injected into the catheter at the back of the balloon. The resultant fluid-filled distal duodenum and proximal jejunum adjacent to the gastric body were identified via a curved linear array echo endoscope (Fujifilm SU-9000, Tokyo, Japan). EUS-guided gastro-enterography with an electrocautery‐enhanced delivery system (Hot AXIOS System, ∅15 mm; Boston Scientific, Marlborough, Massachusetts, USA) was successfully deployed to insert lumen-apposing metal stents through the gastric wall into the jejunal wall ( Fig. 4 ).

The whole procedure took approximately 12 minutes ( Video 1 ). No adverse events were detected during the 3-month follow-up.

An endoscopic ultrasound (EUS)-guided single balloon-occluded gastrojejunostomy bypass was achieved by deploying lumen-apposing metal stents with an electrocautery‐enhanced delivery system through the gastric wall into the jejunal wall under EUS gastro-enterography guidance.Video 1

Endoscopy_UCTN_Code_TTT_1AS_2AG

